# The effects of upper and lower limb exercise on the microvascular reactivity in limited cutaneous systemic sclerosis patients

**DOI:** 10.1186/s13075-018-1605-0

**Published:** 2018-06-05

**Authors:** A. Mitropoulos, A. Gumber, H. Crank, M. Akil, M. Klonizakis

**Affiliations:** 1 Centre for Sport and Exercise Science, Collegiate Campus, Sheffield Hallam University, Collegiate Crescent, Sheffield, S10 2BP UK; 2 Centre for Health and Social Care Research, Sheffield Hallam University, Sheffield, UK; 3 Rheumatology Department, Royal Hallamshire Hospital, Sheffield, UK

**Keywords:** High-intensity interval training, Vascular function, Quality of life

## Abstract

**Background:**

Aerobic exercise in general and high-intensity interval training (HIIT) specifically is known to improve vascular function in a range of clinical conditions. HIIT in particular has demonstrated improvements in clinical outcomes, in conditions that have a strong macroangiopathic component. Nevertheless, the effect of HIIT on microcirculation in systemic sclerosis (SSc) patients is yet to be investigated. Therefore, the purpose of the study was to compare the effects of two HIIT protocols (cycle and arm cranking) on the microcirculation of the digital area in SSc patients.

**Methods:**

Thirty-four limited cutaneous SSc patients (65.3 ± 11.6 years old) were randomly allocated in three groups (cycling, arm cranking and control group). The exercise groups underwent a 12- week exercise program twice per week. All patients performed the baseline and post-exercise intervention measurements where physical fitness, functional ability, transcutaneous oxygen tension (ΔTcpO_2_), body composition and quality of life were assessed. Endothelial-dependent as well as -independent vasodilation were assessed in the middle and index fingers using LDF and incremental doses of acetylcholine (ACh) and sodium nitroprusside (SNP). Cutaneous flux data were expressed as cutaneous vascular conductance (CVC).

**Results:**

Peak oxygen uptake increased in both exercise groups (*p* < 0.01, d = 1.36). ΔTcpO_2_ demonstrated an increase in the arm-cranking group only, with a large effect, but not found statistically significant,(*p* = 0.59, d = 0.93). Endothelial-dependent vasodilation improvement was greater in the arm-cranking (*p* < 0.05, d = 1.07) in comparison to other groups. Both exercise groups improved life satisfaction (*p* < 0.001) as well as reduced discomfort and pain due to Raynaud’s phenomenon (*p* < 0.05). Arm cranking seems to be the preferred mode of exercise for study participants as compared to cycling (*p* < 0.05). No changes were observed in the body composition or the functional ability in both exercise groups.

**Conclusions:**

Our results suggest that arm cranking has the potential to improve the microvascular endothelial function in SSc patients. Also notably, our recommended training dose (e.g., a 12-week HIIT program, twice per week), appeared to be sufficient and tolerable for this population. Future research should focus on exploring the feasibility of a combined exercise such as aerobic and resistance training by assessing individual’s experience and the quality of life in SSc patients.

**Trial registration:**

ClinicalTrials.gov (NCT number): NCT03058887, February 23, 2017.

**Electronic supplementary material:**

The online version of this article (10.1186/s13075-018-1605-0) contains supplementary material, which is available to authorized users.

## Background

Systemic sclerosis (SSc) is an idiopathic systemic autoimmune disease characterized by an ongoing cutaneous and visceral fibrosis, fibroproliferative vasculopathy and immunologic abnormalities [1–4]. The vascular element has an important role in the SSc pathophysiology from early onset to late complications (e.g., pulmonary arterial hypertension and kidney disease). SSc can be distinguished in either limited cutaneous scleroderma (lcSSc) with skin involvement mainly limited to the hands and face; or diffuse cutaneous scleroderma (dcSSc) with skin involvement proximal to the elbows and knees [5]. Blood vessels are directly affected by SSc, as manifested by the diverse clinical complications that take place from the initiation to the propagation of the disease, and have important ramifications on the quality of life (QoL) of patients.

Raynaud’s phenomenon (RP) precedes other clinical manifestations and is observed in over 95% of SSc patients [6]. Evidently, RP is triggered by endothelial injuries in association with dysregulations in the vascular tone [7]. In addition to the imbalance of vascular tone, RP is also associated with structural vascular alterations in small- and medium-sized arteries leading to luminal narrowing. As a result, the blood vessels are unable to compensate for the impairment of blood flow during severe RP attacks and this leads to the so-called ischaemia-reperfusion reactions. These vascular complications may progress to gangrene and digital amputation [8]. Notably, SSc has the highest case-specific mortality of any rheumatic disease being also associated with substantial morbidity [9].

Pharmacological agents (e.g., nifedipine) are commonly used as first-line approach. Although it can be effective and provide pain-relief to patients, the short-term (e.g., oedema, headaches, heart palpitations, dizziness and constipation) and long-term (e.g., heart dysfunction, increased cardiovascular risk) side effects of the medical treatment should also be considered as well as the financial cost of treatment. Therefore, alternative approaches with less side effects and cost implications are warranted [10, 11], with a view to reducing dependency on medication.

Exercise in general and high-intensity interval training (HIIT) specifically could be a useful adjunct therapy for this population. HIIT has come to prominence over the last few years for its effectiveness in inducing greater improvements in vascular function than moderate-intensity continuous training in a number of clinical populations (e.g., heart failure, metabolic syndrome, obesity) [12]. Nevertheless, due to the variation in HIIT protocols, limited evidence exists to support which protocol would be the most effective in SSc patients, although the options are many, based on evidence from other patient populations. For example, a HIIT protocol with short intervals (30 s exercise/30 s passive recovery) may elicit more favourable patient-reported satisfaction/enjoyment levels compared to other longer duration exercise protocols [13]. In chronic heart failure patients, a short duration HIIT protocol (30 s exercise/30 s passive recovery) has demonstrated to be a well-tolerated, preferred protocol with a low perception of effort, patient comfort and with a longer time spent at higher percentage of peak oxygen uptake (V̇O_2peak_) than a longer duration HIIT protocol with active recovery phases [13]. Recent evidence supports this notion; when enjoyment levels in an overweight/obese cohort were examined after a short HIIT protocol and demonstrated that performing a HIIT protocol on a cycle ergometer present on an average 4.5 rating on a 7-point scale [14].

Although we know the potential of HIIT in improving both the micro-and the macro- vascular function in several clinical populations such as heart failure [15] and cardiometabolic disease [16] by using the treadmill and cycle ergometer as modes of exercise, no evidence exists about the mode of exercise that would be more effective on digital microcirculation where the RP attacks are present, such as in SSc patients. Assumptions could be made that utilising an upper-body exercise would potentially be more beneficial for the digital microcirculation rather than lower-body exercise where the working muscles promote the blood flow in the lower limbs. Hence, the effects that may occur by the upper- and lower-limb exercise on digital microcirculation in SSc patients should be examined.

We will attempt to bridge the knowledge gap by assessing the effects of a supervised and individually-tailored exercise programme based on arm cranking (ACE) and cycle ergometry (CE) on microvascular reactivity, aerobic capacity, exercise tolerance and enjoyment levels, as well as on QoL in SSc patients.

## Methods

### Patients

We recruited 34 patients (31 women, 3 men) with lcSSc, defined as per the American College of Rheumatology and European League Against Rheumatism criteria [17], with disease duration between 1 to 10 years. All participants were able to undertake exercise. Patients with pulmonary arterial hypertension, interstitial lung disease, those diagnosed with another inflammatory condition and/ or presenting myositis with proximal muscle weakness were excluded. Moreover, patients with New York Heart Association class 3 or 4, smokers or people who stopped smoking within 4 weeks of screening and women who were pregnant were also not permitted to participate. Eligible patients were recruited from the Rheumatology Department of the Royal Hallamshire Hospital in Sheffield. All patients provided written consent to participate. The regional health research ethics committee for clinical studies approved the protocol. Patients were randomly allocated between the ACE (*n* = 11), CE (*n* = 11) and control (*n* = 12) groups. All the pre- and post-intervention tests were performed at the same time of the day to minimize intra-day variability.

### Procedures

**Fig. 1:**
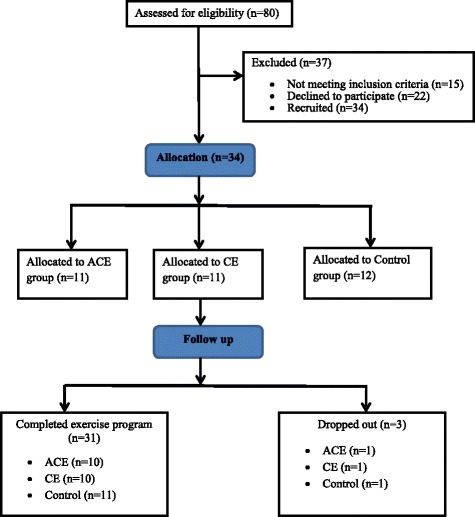
CONSORT flow diagram. *ACE* arm crank ergometer, *CE* cycle ergometer

Baseline assessments, undertaken at first visit, included V̇O_2peak_, anthropometry, functional ability, microvascular reactivity and QoL. V̇O_2peak_ test was performed either on an arm crank ergometer (ACE group) or on a cycle ergometer (CE and control group). Thereafter, patients were randomly allocated to three groups (ACE, CE and control group). The exercise groups (ACE and CE) performed a 12-week exercise programme and the control group did not perform any type of physical activity. All groups were followed up after a 12-week period performing the same measurements as in the baseline. Figure 1 depicts the study’s procedures.

### Anthropometry

The participant’s stature was measured using a Hite-Rite Precision Mechanical Stadiometer. Body weight (kg), body mass index (BMI), fat mass (kg) and lean body mass (kg) segmented in upper and lower limbs were assessed by using bio-electrical impedance analysis (In Body 720, Seoul, Korea). Patients’ demographic characteristics are illustrated in Table 1.

**Table 1:** Demographic data (means ± SD)

	Baseline ACE	Baseline CE	Baseline Control
Age (years)	69.1 ± 9.7	65.1 ± 10	62.2 ± 14.3
Body weight (kg)	69 ± 15.8	66 ± 9.7	73.2 ± 14.8
Body mass index (kg/m^2)^	25.6 ± 4.8	24.5 ± 3.6	27.3 ± 4.0
Stature (cm)	163.7 ± 9.1	164.4 ± 7.9	163.4 ± 6.7
Disease duration (yrs)	7.8 ± 2.3	7.7 ± 2.1	6.3 ± 2.0
Digital ulcers (treatment iloprost infusion)	0/10	0/10	4/11
Raynaud’s treatment	6/10	5/10	8/10
Nifedipine	4/10	4/10	4/10
Sildenafil	2/10	1/10	4/10
Blood pressure treatment	6/10	4/10	4/10
Candesartan	4/10	0/10	1/10
Ramipril	2/10	4/10	3/10

*ACE* arm crank ergometer, *CE* cycle ergometer

### Peak oxygen uptake test

During the cardiopulmonary tests gas exchange was collected and analysed by an online breath-by-breath analysis system (Ultima™, Medical Graphics, Gloucester, UK). Heart rate (HR) was continuously monitored using a Polar heart rate monitor (Polar FS1, Polar Electro, Kemple, Finland) and blood pressure was assessed by the researcher using a manual sphygmomanometer (DuraShock DS54, Welch Allyn, Beaverton, OR, USA) and a stethoscope (Littman Classic II, 3 M, Maplewood, MI, USA). Rating of perceived exertion (RPE) was recorded during the last 10 s of every minute during the exercise test until volitional exhaustion using Borg’s scale [18] 6–20 point. Peak power output (PPO) and test duration was measured in both tests. V̇O_2peak_ defined as the average oxygen consumption was recorded from expiratory samples during the final 30 s of exercise.

### Arm crank test

The arm crank ergometer (Lode BV, Groningen, Netherlands) was adjusted to ensure alignment between the ergometer’s crankshaft and the centre of the patient’s glenohumeral joint. Patients’ sitting position was set up to ensure that the elbows were slightly bent when the arm was outstretched. Patients were instructed to maintain their feet flat on the floor at all times. Due to differences in gender power capabilities, two separate protocols were instructed for men and women. Men commenced at a workload of 30 W and women at 20 W. In both protocols the crank rate was maintained at 70 rev min^− 1^ [19, 20] and power requirements increased as a linear ramp at a rate of 10 W/min and 6 W/min for men and women, respectively [20]. The test commenced with 3 min resting and then 3 min of warm-up (unloaded cranking). RPE ≥ 18 and/or inability to maintain a crank rate above 60 rev min^− 1^ resulted in the termination of the test. After the exercise termination an unloaded bout of 2–3 min exercise at a crank rate below 50 rev min^− 1^ followed allowing for an active recovery period.

### Cycle ergometer test

The cycle ergometer test was performed on an electromagnetic cycle ergometer (Lode Excalibur, Groningen, Netherlands). The test commenced with a 3 min resting period followed by 3 min of unloaded pedalling. Participants were requested to maintain a cycle rate of 60 rev min^− 1^ during the exercise test. The starting load and the concomitant increments were individually calculated according to participants’ estimated physical fitness and Wasserman’s eqs. [21]. RPE ≥ 18 and/or inability to maintain a crank rate above 40–45 rev min^− 1^ resulted in the termination of the test. Following the exercise test 2–3 min of unloaded pedalling was performed to allow for an active recovery period.

### Exercise program

**Fig. 2:**
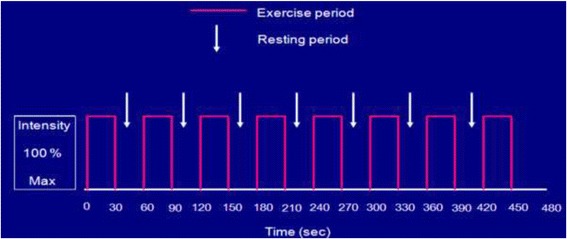
Schematic training protocol

Patients undertook twice-weekly supervised exercise sessions at the Centre of Sport and Exercise Science at Sheffield Hallam University. Each session started with a 5 min warm-up on an arm crank or cycle ergometer depending on the group (involving light aerobic exercise and gentle range of motion exercises). This was followed by HIIT for 30 s at 100% of PPO interspersed by 30 s passive recovery for a total of 30 min (Fig. 2). At the end of the session patients undertook a 5 min cool-down period, involving lower- and upper-limb light intensity aerobic exercise and light stretching. Patients were wearing heart rate monitors throughout the exercise sessions. Heart rate and RPE and effect (see below) were assessed at regular intervals throughout the supervised exercise session.

### Functional ability test

The functional ability was assessed through a six-minute walking test (6MWT). Although the 6MWT lacks organ specificity in SSc, it can provide a valuable outcome parameter and thus, is suggested as a regular assessment in this clinical condition [22]. Patients were instructed to walk as far as possible back and forth on a 10 m corridor for 6 min. They were also instructed to slow down, stop and/or rest as necessary if they got out of breath or became exhausted, but to resume walking as soon as they felt able to. The laps and the total walking distance were recorded on a worksheet.

### Microvascular reactivity

Microvascular function was assessed by laser Doppler Fluximtery and Iontophoresis technique in a temperature-controlled room (22–24 °C). Laser Doppler fluximetry (LDF) electrodes were attached to the dorsal aspect of the reference fingers for acetylcholine (ACh) and sodium nitroprusside (SNP) administration. These were used as indicators of the changes occurring in the endothelial-dependent and -independent vasodilatory function. Heart rate (Sports Tester, Polar, Finland) and blood pressure of the brachial artery (left arm; Dinamap Dash 2500, GE Healthcare, Chicago, IL, USA) were monitored at 5-min intervals throughout the protocol. The two drug delivery electrodes (PF383; Perimed AB, Jarfalla, Sweden) were positioned over healthy-looking skin, approximately 4 cm apart with one containing 100 μL of 1% ACh (Miochol-E, Novartis, Stein, Switzerland.) and the other 80 μL of 1% SNP (Nitroprussiat, Rottapharm, Monza, Italy). ACh was placed over the middle finger between the distal and proximal interphalangeal joints and SNP was placed over the index finger between the metacarpophalangeal and carpometacarpal joints. The incremental iontophoresis protocol for ACh and SNP delivery is described in Klonizakis et al., [23, 24].

### Transcutaneous oxygen pressure (TcpO_2_)

TcpO_2_ measurements were performed during the cardiorespiratory tests using sensors that were non-invasively attached onto the skin and allowed to heat. The sensors induce skin blood capillaries dilatation through heat, which increases the blood flow and results in oxygen diffusion through the skin to the sensor. The sensor measures TcpO_2_ values inwardly through an electrochemical process.

Measurements were performed using the TINA TCM400 TcpO_2_ device (Radiometer, Copenhagen, Denmark). The temperature of the probe was set to 44.5 °C to allow maximal skin vasodilation, thereby decreasing the arterial to skin surface oxygen pressure gradient. Before the exercise test 15–20 min were allowed with the probe attached on the skin for stabilisation of TcpO2 value. After the test the TcpO2 values were automatically corrected according to a temperature of 37 °C by the TINA device. The electrode was placed slightly below the right scapula on the back away from any bone.

Fixation rings were used to hold the probe attached to the skin and this was filled with two small drops of contact fluid before attachment to the sensor. The fluid was then heated causing the subsequent dilatation of the skin. The raw values of the patient’s oxygen perfusion, obtained directly from TcpO2 device were defined (Table 2) as previously described in Wasilewski et al. [25].

**Table 2:** Definitions of TcpO2 quantities

TcpO2 quantity	Definition
Baseline	The arithmetic mean of maximum TcpO2 at rest.
TcpO2_max_	The highest TcpO2 value recorded every minute of exercise or at rest.
Maximum change from baseline (ΔTcpO2_max_)	The outcome of the subtraction of baseline from TcpO2_max_: e.g. TcpO2_max_ - baseline
Changes in transcutaneous oxygen pressure (ΔTcpO2)	The average sum of the change from baseline at rest and exercise period: e.g. (Σ)ΔY1…n) / n) = ΔTcpO2

Δ*TcpO*_*2*_ transcutaneous oxygen tension

### Quality of life

The EQ-5D-5 L was the main outcome used to assess the patients’ quality of life pre- and post-exercise intervention. The EQ-5D-5 L is a generic measure of health state by considering five key dimensions of daily living (mobility, self-care, ability to undertake usual activities, pain, anxiety/depression) [26]. Participants were asked to describe their level of health on each dimension using one of five levels: no problems, slight problems, moderate problems, severe problems, extreme problems. Patients were also asked about to rate their life satisfaction on a scale of zero to ten as well as to rate the RP pain during the last couple of weeks on one to five ascending grading: not at all, slightly, moderately, severely, extremely. Digital ulcers and hospitalization for iloprost infusion and amputations were also recorded.

### Exercise tolerance

The exercise tolerance of HIIT was assessed through measures that were interpreted participants’ perception regarding the exercise intensity, the effect (Additional file 1), the exercise task self-efficacy (Additional file 2), the intentions (Additional file 3) and the enjoyment (Additional file 4). The above data was collected at the first and last exercise session each month in order to examine several time points during the exercise intervention. Specifically, the questionnaires were repeated at the 1st, 8th, 16th, and 24th exercise sessions. The individual questionnaires and the time points that were incorporated during the exercise session are described in Jung et al. [27].

### Statistical analysis

Data analysis was performed using SPSS software (version 23, IBM SPSS, Armonk, NY, USA) and is presented as mean ± SD. Normal distribution of the data and homogeneity of variances were tested using the Shapiro-Wilk and Levene’s test, respectively. The comparison in the anthropometric, physiological and vascular characteristics among the three groups was done through a one-way ANOVA test. Independent t-tests and chi-squared tests were also used to identify the differences between two groups. Effect sizes (Cohen’s d) were calculated wherever the results were statistically significant with 0.2, 0.5, and 0.8 representing small, medium, and large effects respectively [28]. To compare the between group differences using a one-way ANOVA we adjusted the ACE values according to the physiological and anthropometrical responses of CE [29]. Statistical significance was set at *p* ≤ 0.05.

## Results

### Compliance and exercise intensity

Compliance to the12-week exercise programme twice weekly was 92% and 88% for the ACE and CE group respectively, with one drop-out for each exercise group. No exercise-related complications were reported. The average percentage peak HR (%HR_peak_) for each exercise session was 92.1% ± 6.0 for the ACE group and 90.8% ± 7.5 for the CE group. The average rate of perceived exertion (RPE) and effect were 13 ± 1 and + 3 (good) ± 1, respectively, for both exercise groups.

### Oxygen uptake and pressure

Both ACE (0.86 L min^− 1^ d = 0.68) and CE (1.22 ± 0.33 L min^− 1^ d = 0.76) V̇O_2peak_ were significantly greater post-exercise intervention compared to baseline (*p* < 0.01). ACE V̇O_2peak_ (21.9 ± 7.1 ml kg^− 1^ min^− 1^ d = 1.09) improved significantly in comparison to control but not compared to CE group (Table 3).

**Table 3:** Physiological and quality of life outcomes

	ACE (*n* = 10)		CE (*n* = 10)		Control (*n* = 11)	
	Pre	Post	Pre	Post	Pre	Post
ACh CVC	0.14 ± 0.06	0.19 ± 0.08	0.20 ± 0.11	0.26 ± 0.1	0.20 ± 0.08	0.15 ± 0.08
ACh CVC_max_	1.28 ± 0.78	1.56 ± 0.88*	1.49 ± 0.99	1.26 ± 0.52	1.40 ± 0.78	0.82 ± 0.47
ACh T_max_ (sec)	159.4 ± 83	104.1 ± 71.8	172 ± 57.9	119.4 ± 82.9	127.9 ± 51.1	149.9 ± 70.3
SNP CVC	0.15 ± 0.08	0.24 ± 0.14	0.21 ± 0.11	0.25 ± 0.08	0.20 ± 0.09	0.20 ± 0.1
SNP CVC_max_	1.73 ± 2.01	1.88 ± 1.52	1.61 ± 1.21	2.38 ± 1.8	1.70 ± 1.3	1.40 ± 0.56
SNP T_max_ (sec)	161.2 ± 88.5	131.3 ± 77.5	167.4 ± 66.3	138.8 ± 80.5	165.5 ± 56.5	166.9 ± 76.4
ΔTcpO2	2.5 ± 4.0	9.2 ± 12.1	1.56 ± 4.8	1.56 ± 9.5	1.39 ± 3.4	0.89 ± 2.6
ΔTcpO2_max_	11.5 ± 3.9	18.4 ± 16.5	11.7 ± 3.6	13.6 ± 9.6	9.44 ± 7.7	8.0 ± 7.0
V̇O_2peak_ (ml kg^− 1^ min^− 1^)	17.7 ± 4.7	21.9 ± 7.1*	14.6 ± 2.9	18.5 ± 2.8*	14.3 ± 6.9	14.7 ± 6.2
Life satisfaction	6.5 ± 1.6	8.1 ± 1.7***	8.4 ± 1.4*	8.8 ± 1.1***	7.5 ± 1.6	4.9 ± 1.5
Mobility	2.4 ± 1.0	2.3 ± 0.8	1.9 ± 0.9	1.7 ± 1.0	1.9 ± 0.9	2.3 ± 1.2
Self-care	1.1 ± 0.3	1.1 ± 0.3	1.2 ± 0.4	1.0 ± 0.0	1.4 ± 0.9	1.7 ± 1.4
Usual activity	2.3 ± 1.3	1.9 ± 1.1	1.9 ± 1.0	1.6 ± 0.7	1.8 ± 1.0	2.4 ± 1.2
Pain/ discomfort	2.4 ± 1.0	2.3 ± 1.1	2.8 ± 1.1	1.8 ± 0.9	2.4 ± 0.7	2.8 ± 1.2
Anxiety/ depression	1.7 ± 0.8	1.5 ± 0.7	1.6 ± 0.7	1.2 ± 0.4	1.6 ± 0.7	1.9 ± 1.4
Raynaud’s pain	2.4 ± 1.4	1.8 ± 0.6*	2.6 ± 1.5	1.9 ± 1.2*	2.4 ± 0.9	3.1 ± 1.1

Endothelial function presented as cutaneous vascular conductance (CVC). T_max_ is the time taken to reach peak perfusion. *p < 0.05 and ***p < 0.000 compared to the other groups

*ACE* arm crank ergometer, *SNP* sodium nitroprusside, *ΔTcpO*_*2*_ transcutaneous oxygen tension *V̇O*_*2peak*_ peak oxygen uptake

A tendency to improve was also observed in both ΔTcpO2 (*p* = 0.59, d = 0.93) and transcutaneous oxygen tension (ΔTcpO2_max_) (*p* = 0.71, d = 0.80) in ACE group. Although this improvement is not statistically significant the Cohen’s d reveals that the effect size of the change is large (> 0.8) both at rest and during provocation (exercise test).

### Cutaneous vascular conductance (CVC)

No statistically significant differences were observed at baseline between the exercise and control groups (*p* > 0.05). Post-exercise intervention improvements were observed in the ACE group, especially over the control group, while values in CE group were slightly decreased (Table 3).

### Feasibility and tolerance of exercise

ACE showed to be the mode of exercise that will more likely (*p* < 0.05) engage SSc patients to physical activity twice per week (6.9 ± 0.3, d = 1.17) compared to the CE group (6.2 ± 0.79). Moreover, ACE demonstrated to be better (*p* < 0.05) regarding participant’s confidence to perform two bouts per week (95 ± 7%, d = 0.82) than CE (83 ± 19.5%) but not statistically significant. Both exercise modes aggregated a high score of enjoyment levels > 94 out of 119 with an average effect before, during and after the exercise session of + 3 equals to “good”.

### Quality of life and clinical outcomes

The EQ-5D-5 L questionnaire did not demonstrate any significant difference between the groups neither at baseline nor after the completion of the exercise intervention, in any of its five elements. However, both exercise groups reported improved life satisfaction (*p* < 0.000) as well as reduced discomfort and pain of Raynaud’s phenomenon (*p* < 0.05) after the exercise intervention compared to the control group (Table 3). We also reported digital ulcers and hospitalization for iloprost infusion for four out of eleven patients (36.3%) in the control group. One of them proceeded to amputation of the distal phalange of the middle finger in one hand.

## Discussion

Overall, this study is the first to demonstrate that upper-limb aerobic exercise may be able to improve microvascular endothelial-dependent function in the digital area in patients with systemic sclerosis experiencing Raynaud’s phenomenon. Cycling indicated that it might have the potential to decelerate the disease progression in the vasculature (ACh) as the endothelial-dependent vasodilation was slightly decreased. On the other hand, the control group showed a decrease in endothelial-dependent function, which might indicate a disease worsening (Table 3). Pearson’s correlation coefficient (Table 4) indicated that the endothelial improvement in ACE has a trend to correlate with the soft lean and fat-free mass as well as with skeletal muscle mass. Interestingly, ACh showed that is not correlated with ACE V̇O_2peak_, which does not confirm to previous findings that have shown association of endothelial-dependent function with the improvement in aerobic capacity in patients with rheumatoid arthritis [30]. The correlation between the endothelial-dependent function and the lean muscle is a vital evidence for future exercise prescription for this population. Resistance training is capable to increase muscle mass and to improve microcirculation in obese adults [31]. Thus a combination of the current HIIT protocol with resistance training might increase the chances for further improvement in the endothelial function.

**Table 4:** Endothelial-dependent correlations in arm cranking

		Soft lean mass (kg)	Fat-free mass (kg)	Skeletal muscle mass (kg)	V̇O_2peak_ (L min^−1^)	V̇O_2peak_ (ml kg^− 1^ min^− 1^)
ACh CVC_max_	Pearson’s r	0.529	0.520	0.530	0.120	0.220
	sig (2-tailed)	0.116	0.123	0.115	0.740	0.569
	n = 10	10	10	10	10	10

*Ach* acetylcholine, *CVC* cutaneous vascular conductance

### Endothelial-dependent function

Our results indicate that exercise training may improve the microvascular function in SSc patients. This could be largely attributed to a shear-stress-related mechanism. Shear stress is a mechanical reaction of the blood vessel to accommodate the increased blood flow, which activates the potassium channels and facilitates the calcium influx into the endothelial cells. Endothelial nitric oxide synthase (eNOS) activation and expression are triggered by an increase in intracellular calcium [32], promoting nitric oxide (NO) production and thus vasodilation [33]. It is possible that the recurring induction of NOS activity with exercise training decelerates the degradation of NO by free radicals in these conditions [34] or by reducing directly free radical production [35]. A recent systematic review on exercise training and vascular function [12] supports our findings indicating that the antioxidant status is enhanced after HIIT in patients with cardiometabolic disorders [36–38] and thus, the NO bioavailability is improved. Mitranun et al. [38] assessed the effects of interval aerobic exercise training (three times/week for 12 weeks) on endothelial-dependent vasodilation in patients with type 2 diabetes mellitus. The vascular outcomes demonstrated reductions in erythrocyte malondialdehyde and serum von Willebrand factor and increases in plasma glutathione peroxidase and nitric oxide (all *p* < 0.05). Therefore, HIIT seems to improve the microvascular function by reducing oxidative stress markers and enhance the antioxidants as well as the vasodilators in cardiometabolic conditions and potentially in connective tissue diseases such as SSc.

### Vascular remodelling, shear stress and exercise training

Evidence for the time course of functional or structural arterial adaptations to exercise training in humans is limited: Short-term effects of exercise improves NO bioavailability, whereas long-term effects induce changes in vascular remodelling [39], an endothelium and NO-dependent outcome [40].

Prior to this study, we hypothesised that upper limb exercise would be more effective to improve microcirculation in the local regions compared to lower limb exercise; however, the existing evidence supported systemic effects occur after exercise training in the lower limbs [12]. Therefore, we proceeded to a comparison between the upper and lower limbs. Interestingly, this systemic effect was not proved with our study, where the microvascular reactivity in the digital area was improved with arm cranking but not with cycling. Similar to our findings, Klonizakis et al., [41] reported that arm exercise did not have any impact on lower limbs microcirculation in post-surgical varicose-vein patients. It seems that systemic effects of exercise training can only affect the vascular function in the large arteries (e.g. brachial artery) but not the conduit and resistance arteries. Moreover, the mass of muscle engaged in exercise training could play an important role in the systemic effects as studies that utilized handgrip training have not demonstrated contralateral limb remodelling [42–44]. The explanation probably relies on the magnitude and pattern of shear stress, which in turn triggers the release of NO and acts as a main determinant for its bioavailability. It is possible that the induced-shear stress by lower limbs is not sufficient to improve the microcirculation in the acral body parts of the upper limbs. Therefore, the volume of blood flow and the magnitude of shear stress induced by HIIT could account for the local effects of exercise training in the smaller arteries [45, 46].

### Clinical outcome

Inadequate blood flow to living tissue is often a painful experience, threatening the life of the tissue involved. Digital tissue loss not only results in disfigurement and functional disability, it is also the clinical manifestation of an underlying systemic disease process [47]. One of the direct consequences of digital ischaemia is the persistent digital ulcers developing irreversible tissue loss in 30% of patients [48]. In our study four out of eleven patients in the control group developed digital ulcers and required hospitalization for iloprost infusion [49, 50] for a period of 1 to 3 weeks and one patient proceeded to digital amputation of the distal phalange in the middle finger in one hand. Hospitalization is a psychologically-stressful procedure for the patient, which directly affects QoL. The most common side effects of iloprost infusion could be headache, flushing of the skin, nausea, vomiting and sweating. Amputation has been reported to occur in one or more digits due to ischaemia in 20.4% of patients with SSc, 9.2% of which have multiple digit loss [51]. QoL in patients with SSc is adversely affected due to digital ischaemia. Consequently, our protocol has demonstrated that is capable of improving digital ischaemia and preventing disease progression and digital ulcers and thus, improving QoL.

### Transcutaneous oxygen pressure

Although the improvement in oxygen pressure at rest and under provocation (exercise test) was not significant in our study, the effect size of this change was large. This indicates that ACE is able to induce systemic changes in oxygen pressure and vascular function in SSc patients, while the control group showed a slight decrease. It is probable that a higher training load or a larger cohort would have revealed a statistically significant difference between ACE and control group. Evidently, further research is needed to substantiate our findings and explore other training protocols, which will reveal the effects of exercise on skin oxygen pressure, when oxygen demand is higher.

### Quality of life

Both modes of exercise have shown improvement in life satisfaction and reduction in pain or discomfort induced by RP attacks after the exercise training. However, further research is required to confirm the improvement in RP by applying more qualitative measures (e.g. case-specific questionnaires and face-to-face interviews). Exercise tolerance, cardiorespiratory fitness, walking distance, muscle strength and function as well as health-related QoL have been demonstrated to be improved in SSc patients after participation in exercise programmes involving aerobic exercise and aerobic exercise combined with resistance training [52]. Therefore, promoting physical activity for the improvement of QoL in SSc patients should be deemed as one of the priorities for future research.

### Feasibility of HIIT

Our findings demonstrate that HIIT (30 s 100% PPO/ 30 s passive recovery) maintained an average effect of + 3 (“Good”) throughout the exercise training for both modes of exercise. It is also noteworthy that the patient’s effect was similar before, during and after the exercise session which could be explained by the moderate cardiorespiratory stress induced by this protocol. Supportive to this finding is the RPE for both groups which averaged to 13 (“Somewhat hard”), a value which is strongly correlated to anaerobic threshold and low to moderate exercise intensity in a large cohort of adults [53]. Exercise intensity and affective response have presented a negative relationship in inactive and overweight adults and it has been reported that as incremental exercise progresses above the ventilatory threshold, the affective response to exercise becomes more negative [54, 55]. Therefore, a short protocol of HIIT seems to not induce great cardiovascular responses in patients with SSc and that might explain the effect’s stability throughout the session.

The intentions regarding engagement to exercise and the task self-efficacy questionnaires as well as the enjoyment levels of the patients could further substantiate whether HIIT is a feasible mode of exercise in SSc patients. Both modes of exercise demonstrated a strong patient’s confidence to perform two and three bouts of exercise with arm cranking being slightly higher than cycling. Both modes of exercise were enjoyable for the patients, however, arm cranking was found to be significantly higher in the intentions for engagement in two bouts of exercise per week compared to cycling. HIIT is a feasible protocol to be implemented in patients with SSc and ACE is considered more acceptable than CE potentially, because it is a new mode of exercise for this population and that might increase their interest to perform an alternative type of exercise.

### Limitations

The sample size of the current study could be deemed a limitation for the current study but we need to stress that SSc is not a common condition such as cardiometabolic diseases (e.g. hypertension, obesity, diabetes) and we strictly adhered to the pre-defined eligibility criteria to present a consistent and reproducible outcome. Moreover, the ratio between women and men is uneven, with SSc women to men ratio being estimated to be 5.2:1 in northeast England [56].

TcpO2 is a direct value of vascular function as changes at rest mimic the changes in arterial pO_2_ during mild or moderate exercise [57, 58]. However, the time response of these changes is relatively slow (90% time response of TcpO2 being approximately 20 s). Carter and Banham [59] demonstrated that TcpO2 values closely followed those assessed by direct arterial sampling during cardiopulmonary exercise testing with 2 min intervals. We acknowledge that our protocol utilized 1 min intervals until symptomatic limitation of exercise, which might affect the accuracy of TcpO2, however, we need to stress that the utilization of TcpO2 measurement in our study was more of a research interest aiming to evaluate the improvement in vascular function after an exercise programme rather than accurately depicting hypoxemia levels in the arterial wall.

Our patients were only of limited cutaneous systemic sclerosis, where the change in skin thickness is little over time compared to diffuse cutaneous systemic sclerosis (Khanna et al., 2017). Moreover, it is common practice in the NHS clinics to assess mRSS only in patients with dcSSc. Therefore, we did not include this measurement in our study; however, we believe that this does not affect our results, as the categorisation of the patients is clear. Also the autoantibody specificities for SSc patients are not included in our study as this is not a standard clinical practice in the area of Sheffield.

## Conclusions

- Aerobic exercise in general, and HIIT (30 s 100% PPO/30 s passive recovery) specifically, involving the upper limbs may improve the microvascular reactivity through an enhancement of the endothelial-dependent function. Our results correlated well with the lean muscle mass, which indicates that resistance training could be a complementary training element in inducing further improvements in microcirculation.
- Our protocol appears to reduce digital ischaemia risk, which can be the leading cause for further systemic complications and a major factor affecting the quality of life. Exercise is a non-invasive, adjunct treatment with no adverse effects that is well-tolerable by patients with SSc.
- There is a need for large multi-centre, randomised-controlled studies to further establish the effects of exercise on SSc patients.

## Additional files

Additional file 1: Feeling scale (SF). (DOCX 38 kb)

Additional file 2: Exercise task self-efficacy. (DOCX 38 kb)

Additional file 3: Intentions for engagement to exercise. (DOCX 35 kb)

Additional file 4: Physical activity enjoyment scale. (DOCX 48 kb)

## Abbreviations

6MWT: Six-minute walking test
ACE: Arm crank ergometer
Ach: Acetylcholine
BMI: Body mass index
CE: Cycle ergometer
CVC: Cutaneous vascular conductance
dcSSc: Diffuse cutaneous scleroderma
eNOS: Endothelial nitric oxide synthase
HIIT: High-intensity interval training
HR: Heart rate
LDF: Laser Doppler fluximetry
lcSSc: Limited cutaneous scleroderma
NO: Nitric oxide
PPO: Peak power output
QoL: Quality of life
RP: Raynaud’s phenomenon
RPE: Rating of perceived exertion
SNP: Sodium nitroprusside
SSc: Systemic sclerosis
V̇E: Minute ventilation
V̇O_2peak_: Peak oxygen uptake
VT: Tidal volume
ΔTcpO_2_: Transcutaneous oxygen tension
