# Supplementary material for: The effects of upper and lower limb exercise on the microvascular reactivity in limited cutaneous systemic sclerosis patients
Source: Arthritis Res Ther. 2018 Jun 5;20:112. doi: 10.1186/s13075-018-1605-0 (PMC5989435; doi:10.1186/s13075-018-1605-0)
Supplement: Supplementary file 1 — Feeling scale (SF). (DOCX 38 kb) [file 13075_2018_1605_MOESM1_ESM.docx]

**Feeling Scale (FS)**

While participating in exercise, it is common to experience changes in mood. Some individuals find exercise pleasurable, whereas others find it to be unpleasant. Additionally, feeling may fluctuate across time. That is, one might feel good and bad a number of times during exercise. Scientists have developed this scale to measure such responses.

**+5 Very good**

**+4**

**+3 Good**

**+2**

**+1 Fairly good**

**0 Neutral**

**-1 Fairly bad**

**-2**

**-3 Bad**

**-4**

**-5 Very bad**
