# Supplementary material for: The effects of upper and lower limb exercise on the microvascular reactivity in limited cutaneous systemic sclerosis patients
Source: Arthritis Res Ther. 2018 Jun 5;20:112. doi: 10.1186/s13075-018-1605-0 (PMC5989435; doi:10.1186/s13075-018-1605-0)
Supplement: Supplementary file 2 — Exercise task self-efficacy. (DOCX 38 kb) [file 13075_2018_1605_MOESM2_ESM.docx]

**Additional file 2**

**Exercise task self-efficacy**

Please circle the percentage that best describes you in the questions below.

**How confident are you that you can….**

1) perform one bout of exercise a week for the next 4 weeks that is just like the one you completed today?

| 10% | 20% | 30% | 40% | 50% | 60% | 70% | 80% | 90% | 100% |
| --- | --- | --- | --- | --- | --- | --- | --- | --- | --- |
| Not at all |  |  |  |  |  |  |  |  | Extremely confident |

2) perform two bouts of exercise a week for the next 4 weeks that is just like the one you completed today?

| 10% | 20% | | 30% | | 40% | | 50% | | 60% | | 70% | | 80% | | 90% | | 100% | |
| --- | --- | --- | --- | --- | --- | --- | --- | --- | --- | --- | --- | --- | --- | --- | --- | --- | --- | --- |
| Not at all |  | |  | |  | |  | |  | |  | |  | |  | | Extremely confident | |
|  | |  | |  | |  | |  | |  | |  | |  | |  | |  |

3) perform three bouts of exercise a week for the next 4 weeks that is just like the one you completed today?

| 10% | 20% | | 30% | | 40% | | 50% | | 60% | | 70% | | 80% | | 90% | | 100% | |
| --- | --- | --- | --- | --- | --- | --- | --- | --- | --- | --- | --- | --- | --- | --- | --- | --- | --- | --- |
| Not at all |  | |  | |  | |  | |  | |  | |  | |  | | Extremely confident | |
|  | |  | |  | |  | |  | |  | |  | |  | |  | |  |
|  | |  | |  | |  | |  | |  | |  | |  | |  | |  |
