# Supplementary material for: The effects of upper and lower limb exercise on the microvascular reactivity in limited cutaneous systemic sclerosis patients
Source: Arthritis Res Ther. 2018 Jun 5;20:112. doi: 10.1186/s13075-018-1605-0 (PMC5989435; doi:10.1186/s13075-018-1605-0)
Supplement: Supplementary file 3 — Intentions for engagement to exercise. (DOCX 35 kb) [file 13075_2018_1605_MOESM3_ESM.docx]

**Additional file 3**

**Intentions for engagement to exercise**

Please rate the extent to which you agree with the following statements.

1) I intend to engage in the type of exercise I performed today at least 2 times per week during the next month.

| 1 | 2 | 3 | 4 | 5 | 6 | 7 |
| --- | --- | --- | --- | --- | --- | --- |
| Very unlikely | Unlikely | Slight unlikely | Neutral | Slight likely | Likely | Very likely |

2) I intend to engage in the type of exercise I performed today at least 3 times per week during the next month.

| 1 | 2 | 3 | 4 | 5 | 6 | 7 |
| --- | --- | --- | --- | --- | --- | --- |
| Very unlikely | Unlikely | Slight unlikely | Neutral | Slight likely | Likely | Very likely |
