# Supplementary material for: The effects of upper and lower limb exercise on the microvascular reactivity in limited cutaneous systemic sclerosis patients
Source: Arthritis Res Ther. 2018 Jun 5;20:112. doi: 10.1186/s13075-018-1605-0 (PMC5989435; doi:10.1186/s13075-018-1605-0)
Supplement: Supplementary file 4 — Physical activity enjoyment scale. (DOCX 48 kb) [file 13075_2018_1605_MOESM4_ESM.docx]

**Additional file 4**

**Physical Activity Enjoyment Scale**

Please rate how you feel about the exercise you just completed.

Look at the statement, choose the left or right hand side of the statement that **most** represents how you felt about participating in your recent exercise session, then choose a number on your chosen side to show how much you agree with that statement.

 If you don’t agree with either side of the statement choose neutral (4).

 Only circle one number per statement.

 Example: I enjoyed it or I hated it.

 If you mainly enjoyed the exercise session you would circle 2.

|  | Absolutely  agree | Mainly  agree | Somewhat  agree | Neutral | Somewhat  agree | Mainly agree | Absolutely agree |  | |
| --- | --- | --- | --- | --- | --- | --- | --- | --- | --- |
|  | 1 | 2 | 3 | 4 | 5 | 6 | 7 |  |  |
| I enjoyed it |  |  |  |  |  |  |  | **I hated it** |  |
|  | 1 | 2 | 3 | 4 | 5 | 6 | 7 |  |  |
| I felt bored |  |  |  |  |  |  |  | **I felt interested** |  |
|  | 1 | 2 | 3 | 4 | 5 | 6 | 7 |  |  |
| I disliked it |  |  |  |  |  |  |  | **I liked it** |  |
|  | 1 | 2 | 3 | 4 | 5 | 6 | 7 |  |  |
| I found it pleasurable |  |  |  |  |  |  |  | **I found it unpleasurable** |  |
|  | 1 | 2 | 3 | 4 | 5 | 6 | 7 |  |  |
| It was not fun at all |  |  |  |  |  |  |  | **It was a lot of fun** |  |
|  | 1 | 2 | 3 | 4 | 5 | 6 | 7 |  |  |
| I found it energizing |  |  |  |  |  |  |  | **I found it tiring** |  |
|  | 1 | 2 | 3 | 4 | 5 | 6 | 7 |  |  |
| It made me depressed |  |  |  |  |  |  |  | **It made me happy** |  |
|  | 1 | 2 | 3 | 4 | 5 | 6 | 7 |  |  |
| It was very pleasant |  |  |  |  |  |  |  | **It was very unpleasant** |  |
|  | **Absolutely**  **agree** | **Mainly**  **agree** | **Somewhat**  **agree** | **Neutral** | **Somewhat**  **agree** | **Mainly agree** | **Absolutely agree** |  | |
|  | 1 | 2 | 3 | 4 | 5 | 6 | 7 |  |  |
| I felt good physically while doing it |  |  |  |  |  |  |  | **I felt bad physically while doing it** |  |
|  | 1 | 2 | 3 | 4 | 5 | 6 | 7 |  |  |
| It was very invigorating |  |  |  |  |  |  |  | **It was not at all invigorating** |  |
|  | 1 | 2 | 3 | 4 | 5 | 6 | 7 |  |  |
| I was very frustrated by it |  |  |  |  |  |  |  | **I was not at all frustrated by it** |  |
|  | 1 | 2 | 3 | 4 | 5 | 6 | 7 |  |  |
| It was very gratifying |  |  |  |  |  |  |  | **It was not at all gratifying** |  |
|  | 1 | 2 | 3 | 4 | 5 | 6 | 7 |  |  |
| It was very exhilarating |  |  |  |  |  |  |  | **It was not at all exhilarating** |  |
|  | 1 | 2 | 3 | 4 | 5 | 6 | 7 |  |  |
| It was not at all stimulating |  |  |  |  |  |  |  | **It was very stimulating** |  |
|  | 1 | 2 | 3 | 4 | 5 | 6 | 7 |  |  |
| It gave me a strong sense of accomplishment |  |  |  |  |  |  |  | **It did not give any sense of accomplishment** |  |
|  | 1 | 2 | 3 | 4 | 5 | 6 | 7 |  |  |
| It was very refreshing |  |  |  |  |  |  |  | **It was not at all refreshing** |  |
|  | 1 | 2 | 3 | 4 | 5 | 6 | 7 |  |  |
| I felt as though I would rather be doing something else |  |  |  |  |  |  |  | **I felt as though there was nothing else I would rather doing** |  |
